# A Practical Model for Predicting Esophageal Variceal Rebleeding in Patients with Hepatitis B-Associated Cirrhosis

**DOI:** 10.1155/2023/9701841

**Published:** 2023-08-03

**Authors:** Linxiang Liu, Yuan Nie, Qi Liu, Xuan Zhu

**Affiliations:** ^1^Department of Gastroenterology, The First Affiliated Hospital of Nanchang University, Nanchang, Jiangxi, China; ^2^Jiangxi Clinical Research Center for Gastroenterology, Nanchang, Jiangxi, China

## Abstract

**Background:**

Variceal rebleeding is a significant and potentially life-threatening complication of cirrhosis. Unfortunately, currently, there is no reliable method for stratifying high-risk patients. Liver stiffness measurements (LSM) have been shown to have a predictive value in identifying complications associated with portal hypertension, including first-time bleeding. However, there is a lack of evidence to confirm that LSM is reliable in predicting variceal rebleeding. The objective of our study was to evaluate the ability of generating a extreme gradient boosting (XGBoost) algorithm model to improve the prediction of variceal rebleeding.

**Methods:**

This retrospective analysis examined a cohort of 284 patients with hepatitis B-related cirrhosis. XGBoost models were developed using laboratory data, LSM, and imaging data to predict the risk of rebleeding in the patients. In addition, we compared the XGBoost models with traditional logistic regression (LR) models. We evaluated and compared the two models using the area under the receiver operating characteristic curve (AUROC) and other model performance parameters. Lastly, we validated the models using nomograms and decision curve analysis (DCA).

**Results:**

During a median follow-up of 66.6 weeks, 72 patients experienced rebleeding, including 21 (7.39%) and 61 (21.48%) patients who rebleed within 6 weeks and 1 year, respectively. In brief, the AUC of the LR models in predicting rebleeding at 6 weeks and 1 year was 0.828 (0.759–0.897) and 0.799 (0.738–0.860), respectively. In contrast, the accuracy of the XGBoost model in predicting rebleeding at 6 weeks and 1 year was 0.985 (0.907–0.731) and 0.931 (0.806–0.935), respectively. LSM and high-density lipoprotein (HDL) levels differed significantly between the rebleeding and nonrebleeding groups, with LSM being a reliable predictor in those models. The XGBoost models outperformed the LR models in predicting rebleeding within 6 weeks and 1 year, as demonstrated by the ROC and DCA curves.

**Conclusion:**

The XGBoost algorithm model can achieve higher accuracy than the LR model in predicting rebleeding, making it a clinically beneficial tool. This implies that the XGBoost model is better suited for predicting the risk of esophageal variceal rebleeding in patients.

## 1. Introduction

Acute hepatitis B is a major health burden, with an estimated 240 million chronic carriers of hepatitis B virus (HBV) surface antigen (HBsAg) worldwide and 815,000 deaths annually due to its complications [1, 2]. Cirrhosis is the end stage of various chronic liver diseases, and esophageal variceal bleeding in cirrhosis is the main life-threatening complication of the decompensated phase. However, first variceal bleeding occurs at a rate of 10–15% per year, and recurrent bleeding occurs at a rate of up to 60% per year [3], which is enough to warrant attention to variceal rebleeding.

Thanks to advanced noninvasive approaches, such as transient elastography, it is possible to conveniently and efficiently determine the degree of liver fibrosis in patients [4]. In addition, there was a good correlation between liver stiffness measurements and portal hypertension [5, 6]. Furthermore, portal hypertension is a consequence of cirrhosis and is an important determinant of the course and prognosis of esophageal varices [7, 8]. However, there are still gaps in the use of noninvasive means to predict patients with hepatitis B cirrhosis experiencing recurrent bleeding.

Modern medicine faces the challenge of using available knowledge to solve clinical problems consisting of sophisticated data. Moreover, the interactions between independent risk factors are nonlinear, and it is relatively inaccurate to analyze the prognostic performance of different factors using traditional linear discriminations [9]. Extreme gradient boosting (XGBoost) is a machine learning technique with the remarkable features of efficient and flexible handling of missing data and combining weak prediction models into accurate prediction models [10]. The algorithm relies on combining predictions from a collection of weak decision and regression trees, which are sequentially added to the model to maximize prediction performance and minimize model complexity. The algorithm is based on predictions from a collection of weak decision and regression trees, and these results are added to the model to maximize prediction performance and minimize model complexity. However, the ability of the XGBoost algorithm in predicting variceal rebleeding in cirrhosis is not yet clear.

The increase in the computing power of smart terminals and the spread of information technology has created the potential for using machine learning models in regular practice. This study aimed to construct an XGBoost model for predicting esophageal variceal rebleeding in cirrhosis and to compare it with the traditional logistic regression model. It also provides a preliminary insight into the clinical features of patients who may experience rebleeding.

## 2. Methods

### 2.1. Study Cohort

This study was a retrospective study of patients with hepatitis B-related cirrhosis hospitalized at the First Affiliated Hospital of Nanchang University between September 2017 and October 2020. The inclusion criteria for patients were as follows: (1) those aged ≥18, (2) those with positive hepatitis B surface antigen and diagnosed with cirrhosis by liver biopsy or imaging examinations together with clinical features, (3) those who first bled in the past and received secondary prevention of variceal rebleeding, (4) and those who had a liver transient elastography measurement before the next episode of variceal bleeding. The exclusion criteria included the following: (1) those with a diagnosis of HCC at inclusion or during the first 6 months of follow-up, (2) those with known HIV, (3) those whose first bleeding is nonesophagogastric vein bleeding under digestive endoscopy, (4) those with history of liver transplantation, (5) those who had a combination with other types of liver disease, (6) the patient had a large amount of ascites that affects the performed liver stiffness measurements, and (7) those who had severe heart and lung disease. The treatment of the included patients was individualized according to the Baveno VII standards [11]. The study protocol was approved by the Institutional Ethics Committee of the First Affiliated Hospital of Nanchang University.

### 2.2. Secondary Prevention, Liver Stiffness Measurement, Clinical Data Collection, and Outcome

Specific details of the secondary prevention practices, liver stiffness measurements, and clinical data collection are provided in the supplementary materials (available here).

The primary outcome was a rebleeding event due to esophageal variceal. Patient survival and special procedures such as TIPS will also be recorded during follow-up periods. The longest follow-up period is 3 years.

### 2.3. Strategies for Model Development

For traditional logistic regression models, we use univariate and multivariate logistic regressions to identify the modeling variables and construct nomograms from the variables. Constructing a nomogram is a method of visualizing the selected features, which intuitively displays the contribution level and mutual cumulative relationship of variables to the outcome and can predict the probability of the outcome. LR model evaluation is done using the area under ROC, C-index, and calibration curve.

The selection of features for the XGBoost model was determined based on clinical importance, scientific knowledge, and previous publications in similar articles. To prevent model overfitting, patients in the cohort were randomly assigned to the derivation set in a ratio of 70%, and the remaining 30% of patients were randomly divided into the validation and test sets in a 1 : 1 ratio. Before constructing the XGBoost model, the data were normalized and the categorical variable data were processed using one-hot coding. The XGBoost model contains various hyperparameters that need to be tuned to the dataset to improve its performance. We perform a grid search on these hyperparameters to find the best combination. To better explain the XGBoost model, the SHapley Additive exPlanations (SHAP) [12] score was estimated to explain the XGBoost predictions. SHAP plots are drawn to represent the contribution of individual predictors to the final model.

Finally, we use AUC, sensitivity, specificity, accuracy, precision, recall, and *F*1 to evaluate model performance and compare between LR and XGBoost models. To identify the net benefit of both models in clinical practice, the LR and XGBoost models were evaluated using a clinical decision curve (CDA).

### 2.4. Statistical Analysis

Continuous variables are shown as the mean and standard deviation (SD) or median and interquartile range (IQR), while categorical variables are shown as frequencies (%). We tested whether the explanatory variable had an interaction and found no significant interactions within the included variables. Student's *t*-tests or Mann–Whitney *U*-test were performed for group comparisons. The diagnostic accuracy of rebleeding was assessed by receiver operating characteristic (ROC) analysis. Areas under the ROC curves (AUCs) were compared by the method of DeLong et al. All levels of significance were set at a two-sided 5% level. All analyses were performed using SPSS 25.0 IBM (IBM Corp., Armonk, NY, USA) and R 3.5.2 (R Project for Statistical Computing, Vienna, Austria). The *R* statistical packages tidyverse, pROC, rms, compareGroups, caret, XGBoost, SHAPforXGBoost, and rmda were used to model construction and statistical analysis.

## 3. Results

### 3.1. Baseline Characteristics

**Figure 1:**
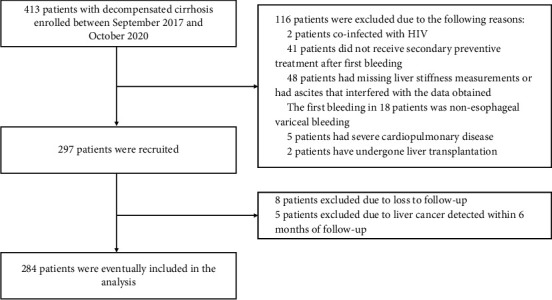
The flowchart of our study.

A total of 284 patients with hepatitis B-related cirrhosis patients who received secondary prevention of esophageal variceal rebleeding and liver stiffness measurement between 2017 and 2020 were included in this study.  Figure 1 shows a flowchart depicting the subject selection procedure, and Table 1 shows the baseline characteristics of the cohort population.

In a median follow-up time of 66.6 weeks, 72 (25.4%) patients presented with endoscopically confirmed rebleeding. In the whole cohort, the majority were male patients, accounting for 68.7%. Their liver stiffness measurements and portal vein diameter were increased compared to the normal population with a median of 14.2 kPa, 1.5 cm. 98 patients, or 34.5% of the cohort, were not taking NSBB drugs because of poor adherence or drug intolerance. In terms of blood cell analysis, the mean values of hemoglobin, white blood cell count, and platelet count in the cohort were 99.4 g/L, 4.33 × 10^9^/L, and 93.3 × 10^9^/L, respectively. In terms of liver function assessment, the majority of patients had a Child–Pugh grade *A*, accounting for 68.7%. Child–Pugh grades *B* and *C* accounted for 26.4% and 4.9%, respectively. In addition, the median MELD score for this cohort of patients was 9.87.

Of the patients first hospitalized, 118 patients had symptoms of hypovolemia that did not progress to nonshock and improved with rehydration. 6 patients presented with stage II or less hepatic encephalopathy and their symptoms were corrected before discharge. 5 patients presented with signs and symptoms of spontaneous peritonitis during hospitalization and confirmed by blood culture or laparotomy. 4 patients completed TIPS before rebleeding, and 1 patient underwent liver transplantation.

### 3.2. Clinical Baseline Comparison between 6 Weeks and 1 Year

To further investigate the differences in baseline clinical characteristics between patients in the esophageal variceal rebleeding group and those in the nonrebleeding group, we compared the baseline differences between patients at two-time points (Table 1). In terms of rebleeding within 6 weeks, clinical baseline variables such as patient follow-up time, liver stiffness values, portal vein diameter, use of NSBB drugs, hemoglobin, platelet count, glutamate transaminase, alkaline phosphatase, glutamine aminotransferase, cholesterol, and LDL differed between the two groups. Similarly, in patients who rebled within 1 year, their clinical baseline variables such as time to follow-up, liver stiffness measurements, portal vein diameter, use of NSBB drugs, hemoglobin, white blood cell count, platelet count, glutamyl transpeptidase, and low-density lipoprotein differed in the two groups.

Notably, at two different time points, clinical baseline variables such as liver stiffness measurements and portal vein diameter were higher in the rebleeding group than in the nonrebleeding group. However, clinical variables such as follow-up time, the proportion of patients on NSBB drugs, hemoglobin, platelet count, glutamyl transpeptidase, and LDL were lower in the rebleeding group than in the nonrebleeding group.

### 3.3. Development and Validation of Logistic Regression Models

We investigated clinical variables associated with variceal rebleeding within the 6 weeks in a univariate logistic regression analysis using enter methods to include all clinical baseline variables. This process identified 9 clinical variables such as liver stiffness measurements, portal vein diameter, use of NSBB drugs, hemoglobin, platelet count, alkaline phosphatase, cholesterol, and LDL associated with variceal rebleeding. Then, we included the abovementioned 9 variables in a multivariate logistic regression and identified 3 variables, the use of NSBB drugs (OR: 0.170 (0.062–0.470), *P*=0.01), hemoglobin (OR: 0.979 (0.960–0.998), *P*=0.029]), and platelets (OR: 0.985 (0.971–0.999), *P*=0.032), as independent risk factors for variceal bleeding (Table 2).

A similar approach was applied to the 1-year observation points. 7 variables, BMI, liver stiffness measurement, portal vein diameter, use of NSBB drugs, hemoglobin, white blood cell count, and platelet count, were identified in a univariate logistic regression analysis. Multifactorial logistic regression analysis identified BMI (OR: 1.146 (1.031–1.273), *P*=0.011), liver stiffness measurements (OR: 1.042 (1.011–1.075), *P*=0.008), use of NSBB drugs (OR: 0.274 (0.127–0.482), *P* < 0.001), hemoglobin (OR: 0.976 (0.963–0.998), *P* < 0.001), and platelet count (OR: 0.989 (0.982–0.996), *P* < 0.001) as independent risk factors for rebleeding in patients within 1 year (Table 2).

**Figure 2:**
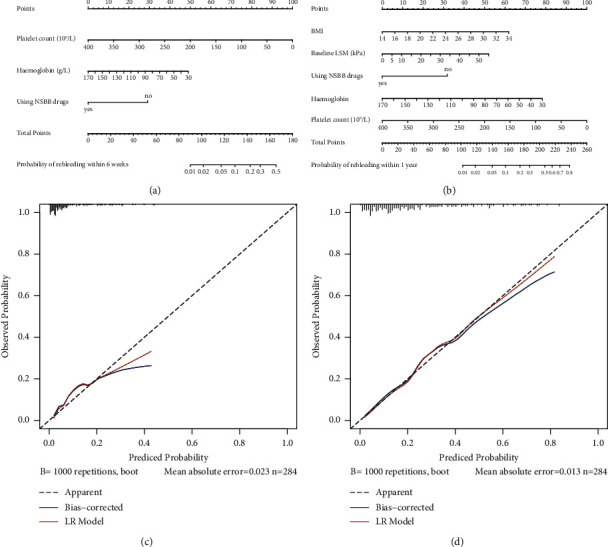
Nomogram and calibration curve for logistic regression model construction: (a) nomogram developed for logistic regression model predicting rebleeding within 6 weeks, (b) nomogram developed for logistic regression model predicting rebleeding within 1 year, (c) calibration curve for predicting rebleeding within 6 weeks, and (d) calibration curve for predicting rebleeding within 1 year.

Based on the independent risk factors obtained above, we developed the nomograms (Figures 2(a) and 2(b)). Evaluating this nomogram using the *R*^2^ and *C*-index and the results showed an *R*^2^ of 0.228 and a *C*-index of 0.828 (0.761–0.896) for the model in predicting rebleeding within 6 weeks. Similarly, in the nomogram predicting rebleeding within 1 year, the *R*^2^ and *C* indices were 0.286 and 0.799 (0.738–0.859), respectively. To further evaluate the model, the accuracy of the model and potential model overfit were assessed by bootstrap validation with 1000 resamplings, the 50-sample bootstrapped calibration plot for the prediction of 6 weeks rebleeding rate and 1-year rebleeding rate (Figures 2(c) and 2(d)). The calibration plots demonstrated excellent consistency between the actual rebleeding rate and the nomogram prediction.

### 3.4. Development and Validation of XGBoost Models

**Figure 3:**
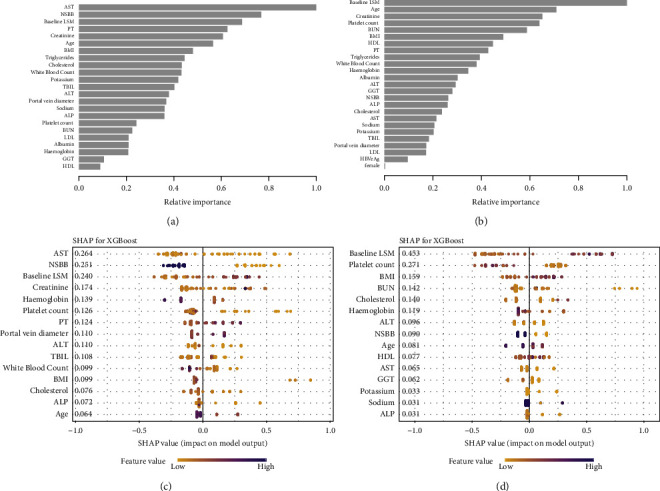
Factors effect size: (a, b) importance evaluation of the XGBoost model for predicting rebleeding within 6 weeks and 1 year. Histogram describes the proportion of factoric importance of different predictors in the model. (c, d): The SHapley Additive exPlanations (SHAP) summary plots show the effect of individual predictors on the output of the XGBoost model for predicting rebleeding within 6 weeks and within 1 year, respectively, as well as the total SHAP score. Diversion on *x*-axis represents impact on model output, with colors used to represent low (yellow) to high (purple) value of predictors.

We developed XGBoost models for rebleeding within 6 weeks and rebleeding within 1 year. In the model for predicting rebleeding within six weeks, the top five relative importance features were aspartate aminotransferase, use of NSBB drugs, liver stiffness measurement, prothrombin time, and blood creatinine level. Similarly, in the model predicting rebleeding within 1 year, the top five relatively important features were liver stiffness measurements, age, blood creatinine, platelet count, and urea nitrogen levels (Figures 3(a) and 3(b)). SHAP analysis values were calculated to compute the contribution of each parameter to the performance of the prediction model. It showed that the top five features were aspartate aminotransferase, use of NSBB drugs, liver stiffness measurements, blood creatinine, and hemoglobin levels in the 6-week model. The top five SHAP value features were liver stiffness measurements, platelet count, BMI, urea nitrogen, and cholesterol levels in the 1-year model (Figures 3(c) and 3(d)).

**Figure 4:**
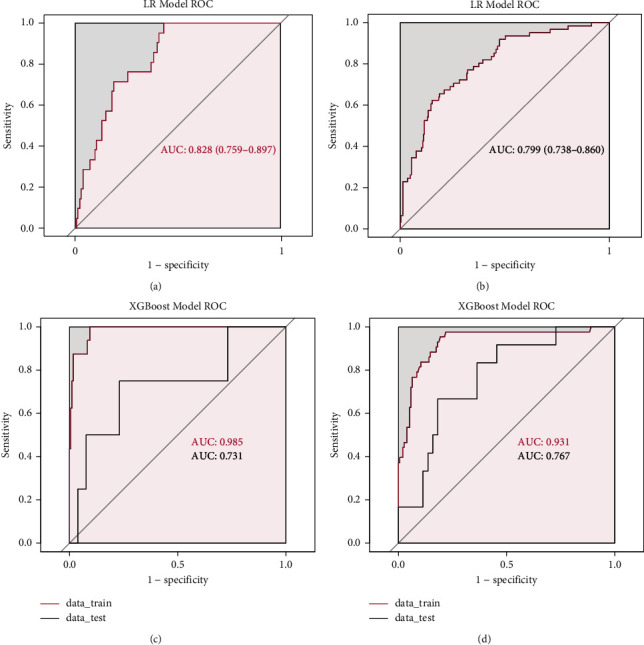
Evaluation of the predictive LR and XGBoost models: (a) receiver operating characteristic curve (ROC) of LR model for predicting rebleeding within 6 weeks. (b) Receiver operating characteristic curve (ROC) of LR model for predicting rebleeding within 1 year. (c) Receiver operating characteristic curve (ROC) of XGBoost model for predicting rebleeding within 6 weeks. (d) Receiver operating characteristic curve (ROC) of XGBoost model for predicting rebleeding within 1 year.

Based on these results, we plotted the ROC curves of this model at different follow-up time points in the training and test sets to evaluate the accuracy of the model. In predicting the rebleeding XGBoost model within six weeks, the AUCs of the XGBoost model in the training and test sets were 0.985 (0.907–1.000) and 0.731 (0.705–0.769), respectively. Similarly, in predicting the rebleeding XGBoost model in 1 year, the AUCs of the XGBoost model in the training and test sets were 0.931 (0.806–0.953) and 0.767 (0.667–0.818), respectively (Figures 4(c) and 4(d)).

### 3.5. Models' Performance Comparisons and Optimal Models' Analysis

In the model development and validation phase, the traditional logistic regression model showed good discriminatory power in different follow-up time points with AUCs of 0.828 (0.759–0.897) and 0.799 (0.738–0.860), respectively (Figures 4(a) and 4(b)). However, the XGBoost model has a better differentiation ability than the traditional logistic regression model with AUCs of 0.985 (0.907–0.731) and 0.931(0.806–0.935), respectively (Figures 4(c) and 4(d)). Other parameters used to evaluate the model such as sensitivity, specificity, accuracy, precision, recall, and *F*1 are detailed in the Table 3.

**Figure 5:**
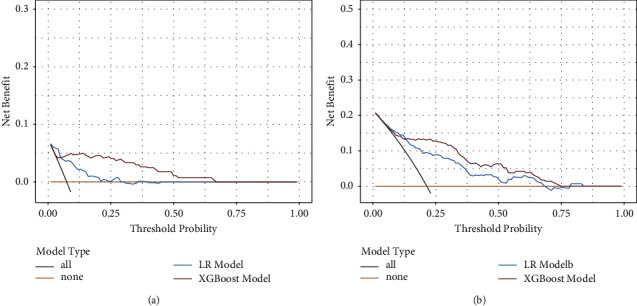
Decision curve analysis (DCA) of LR and XGBoost prediction models. *x* axis indicates the threshold probability of critical care outcome and *y* axis indicates the net benefit. Red solid line = XGboost model; blue solid line = LR model. Its net benefit is larger in XGBoost than in the range of the LR model: (a) DCA shows a comparison of models predicting rebleeding within 6 weeks. (b) DCA shows a comparison of models predicting rebleeding within 1 year.

To visualize the abovementioned results, we developed a nomogram based on traditional logistic regression models to predict rebleeding within 6 weeks and 1 year. Plotting the DCA curves for the logistic regression model and the XGBoost model (Figure 5), it can be visually learned that the net benefit of the XGBoost model is consistently higher than that of the logistic regression model at both time points, which means the XGBoost model is the optimal and the logistic regression model inferior.

## 4. Discussion

In this study, we developed the traditional logistic regression model and the XGBoost algorithm model using common clinical indicators and our study has several novel contributions. For the first time, our study included the XGBoost algorithms' model for comprehensive analyses and compared their predictive performance with the traditional logistic regression model. Whether it is evaluated from the model performance parameters or from the DCA curve to evaluate the patient's net benefit, the results suggest that the XGBoost algorithm model performs better than the traditional LR model. Such results are promising, and this model has the potential to be integrated into electronic medical records and made available in healthcare settings.

In an LR model to predict rebleeding within 6 weeks and 1 year, the presence or absence of NSBB used, platelet count, and hemoglobin were established as common independent risk factors. The guidelines recommend the use of NSBB drugs for secondary prevention of variceal rebleeding [11]. NSBB drugs have been reported not only in secondary prophylaxis but also in the prevention of primary variceal bleeding [13]. Of the predictors of esophageal varices and variceal bleeding in patients with acute upper gastrointestinal bleeding, platelets appear to differentiate between patients with and without esophageal varices [14] and predict patient mortality and rebleeding rates [15]. Interestingly, hemoglobin levels predicted rebleeding in patients at 6 weeks and 1 year. On the one hand, splenic phagocytic activity may lead to anemia and leukopenia in cirrhotic splenomegaly, and other more intricate factors lead to thrombocytopenia [16]. On the other hand, Piano et al. [17] found that baseline hemoglobin levels were an independent risk factor for the development of ACLF. The underlying pathophysiological mechanism can be explained by the fact that low hemoglobin concentrations may further reduce peripheral oxygen delivery, either directly and/or by further impairing macrovascular dysfunction, thereby exacerbating the development of organ failure.

Currently, LSM are promising predictors of progression of compensated cirrhosis to decompensation and predictors of progression of decompensated cirrhosis and are widely validated worldwide [11, 18–20]. Mechanistically, LSM has a good consistency with the degree of liver tissue fibrosis and portal pressure, while PH is the result of liver cirrhosis and is an important determinant of EVB disease course and prognosis [21]. Previous studies [19] have indicated that LSM predicts survival in NAFLD. For that matter, similar results were obtained in our study, where LSM predicted rebleeding over a relatively long time, within one year, in the traditional logistic regression model and the XGBoost algorithm model. Interestingly, LSM was not an independent risk factor in the LR model for predicting rebleeding within 6 weeks. A potential explanation is that LSM is positively related to the degree of liver fibrosis, but it is not a simple linear relationship [22]. In logistic regression analysis, handling such variables and screening is indeed inferior to machine learning models.

To facilitate the implementation and interpretation of the XBGoost model in clinical practice, SHAP analysis, which is a new way to describe the contribution of the predictor's value to the overall prediction of an individual in the XGBoost model, was used for this model. In the XGBoost model for predicting rebleeding within 6 weeks, AST and creatinine levels were both presents in the first five items of the SHAP analysis. The abovementioned results suggest that patients with liver [23] and kidney injury [24] are more likely to have rebleeding within 6 weeks. From another perspective, the use of AST with platelet ratio index (APRI) for the noninvasive diagnosis of clinically significant portal hypertension and esophageal varices has been reported for a long time [25], implying that this parameter plays an important role in predicting rebleeding. In the XGBoost model for predicting rebleeding within 1 year, LSM was the feature that contributed the most to predicting rebleeding. This result is not astonishing, as LSM is not only associated with a good correlation with HVPG [26] but also with its ability to predict liver failure in patients with cirrhosis [17, 27].

In our study, when comparing patients in the bleeding group with those in the nonbleeding group at baseline, LDL levels were found to be significantly lower in patients in the bleeding group than in the nonbleeding group. Xiao et al. [28] found that reduced serum LDL levels were an independent risk factor for survival in patients with HBV-associated ACLF. In this regard, our study expands the predictive disease spectrum of LDL and reveals the potential value of LDL not only in ACLF but also in cirrhotic rebleeding. One of the mechanisms of variceal bleeding in cirrhosis can be explained by inflammation and intestinal bacterial translocation [29]. Previous studies suggest that lipoproteins are required to bind endotoxins caused by intestinal bacterial translocation and to reduce the systemic release of proinflammatory cytokines [30]. Therefore, a decrease in LDL levels and an increase in systemic inflammation in patients are not inconsistent with a tendency to develop variceal bleeding. In addition to this, reduced LDL levels may also simply be the result of liver failure, which is the main source of LDL.

A noteworthy finding in our study is that the features included in the XGBoost model and the logistic regression model show consistency, indicating that the superior performance of the XGBoost model is significant, although the two models fit well and performance may differ. The strengths of this study lie mainly in the first use of the XGBoost model to predict esophageal variceal rebleeding in patients with hepatitis B-related cirrhosis, which was compared with conventional regression analysis and validated by calibration curves and DCA curves. We acknowledge other limitations of our study: first, potential bias may occur due to data from a single center, although our unit is the largest health care facility in our region; second, the proposed model was not validated by other centers or databases; and third, no follow-up measurements of patients' LSM were made during follow-up, as some studies have reported that the value of change in LSM predicts prognosis of patients with postviral hepatitis cirrhosis [31]. Even so, we believe that the proposed model may help us to further understand the prognosis of such patients.

In conclusion, this study shows that machine learning based on the XGBoost algorithm is indeed superior to traditional logistic regression. Meanwhile, LSM proved to be a promising parameter for predicting variceal rebleeding in patients. This would mean transplanting the XGBoost model into an electronic patient management system to scientifically predict the risk of rebleeding and provide personalized care to patients.

## Figures and Tables

**Table 1:** Baseline characteristics of the cohort and baseline comparisons between the nonbleeding and rebleeding groups.

	Baseline	Rebleeding within 6 weeks			Rebleeding within 1 year		
		No	Yes	*P*. overall	No	Yes	*P*. overall
		*N* = 263	*N* = 21		*N* = 223	*N* = 61	
Follow-up (weeks)	66.6 (24.3–135)	90.3 (66.0)	4.40 (1.59)	**<0.001**	102 (64.8)	17.8 (14.8)	**<0.001**
Age (years)	52.24 (12.89)	52.4 (13.1)	50.5 (9.13)	0.383	52.9 (13.1)	50.0 (11.8)	0.108
Gender				0.597			0.848
Male	195 (68.7%)	179 (68.1%)	16 (76.2%)		152 (68.2%)	43 (70.5%)	
Female	89 (31.3%)	84 (31.9%)	5 (23.8%)		71 (31.8%)	18 (29.5%)	
BMI	22.0 (3.07)	21.9 (3.03)	22.2 (3.61)	0.736	21.8 (2.98)	22.6 (3.33)	0.11
Baseline LSM (kPa)	14.2 (10.6–19.2)	13.8 (10.4–19.0)	18.3 (11.8–23.8)	**0.024**	13.4 (10.0–17.7)	18.8 (13.4–22.9)	**<0.001**
Portal vein diameter (cm)	1.50 (1.30–1.60)	1.50 (1.30–1.60)	1.60 (1.50–1.70)	**0.038**	1.50 (1.30–1.60)	1.50 (1.40–1.70)	**0.028**
PVT				0.731			0.384
No	249 (87.7%)	231 (87.8%)	18 (85.7%)		198 (88.8%)	51 (83.6%)	
Yes	35 (12.3%)	32 (12.2%)	3 (14.3%)		25 (11.2%)	10 (16.4%)	
Using NSBB drugs				**0.001**			**<0.001**
No	98 (34.5%)	83 (31.6%)	15 (71.4%)		64 (28.7%)	34 (55.7%)	
Yes	186 (65.5%)	180 (68.4%)	6 (28.6%)		159 (71.3%)	27 (44.3%)	
Hemoglobin (g/L)	99.4 (27.6)	100 (27.7)	86.0 (24.0)	**0.015**	102 (27.5)	88.2 (25.2)	**<0.001**
White blood count (10^9^/L)	4.33 (2.25)	4.38 (2.25)	3.71 (2.25)	0.202	4.55 (2.21)	3.53 (2.22)	**0.002**
Platelet count (10^9^/L)	93.3 (66.2)	96.3 (67.1)	56.0 (37.7)	**<0.001**	101 (70.2)	64.9 (37.9)	**<0.001**
PT (second)	14.0 (3.44)	13.9 (3.50)	14.5 (2.59)	0.399	13.9 (3.70)	14.2 (2.25)	0.48
Albumin (g/L)	35.1 (6.07)	35.1 (6.06)	35.0 (6.39)	0.954	35.0 (6.18)	35.2 (5.70)	0.818
TBIL (*μ*mol/L)	19.2 (12.8–29.6)	19.0 (12.8–29.6)	20.0 (13.3–28.5)	0.774	18.5 (12.3–31.1)	20.2 (13.9–29.5)	0.611
ALT (U/L)	24.5 (16.0–41.5)	25.0 (17.0–43.5)	17.0 (14.0–28.0)	0.094	25.0 (17.0–44.0)	22.0 (16.0–35.0)	0.192
AST (U/L)	35.0 (26.0–52.2)	35.0 (27.0–53.0)	26.0 (19.0–39.0)	**0.043**	35.0 (27.0–53.5)	32.0 (23.0–49.0)	0.096
ALP (U/L)	88.0 (62.8–130)	89.0 (64.0–134)	72.0 (50.0–96.0)	**0.025**	90.0 (64.5–136)	83.0 (56.0–113)	0.068
GGT (U/L)	32.0 (19.0–74.2)	34.0 (20.0–81.5)	21.0 (13.0–33.0)	**0.007**	34.0 (20.0–90.0)	27.0 (18.0–52.0)	**0.039**
BUN (mmol/L)	5.05 (4.00–6.43)	5.00 (4.00–6.35)	5.70 (4.00–6.50)	0.681	5.10 (4.00–6.55)	5.00 (3.70–6.30)	0.402
Creatinine (*μ*mol/L)	65.6 (55.7–77.9)	65.4 (55.8–77.2)	67.8 (51.1–88.9)	0.885	65.5 (56.4–78.2)	65.7 (52.2–75.5)	0.329
Sodium (mmol/L)	139 (5.12)	137 (15.2)	139 (6.21)	0.269	137 (16.3)	137 (6.65)	0.844
Potassium (mmol/L)	3.88 (0.53)	5.91 (16.5)	3.95 (0.67)	0.057	5.82 (16.5)	5.57 (13.5)	0.905
Triglycerides (mmol/L)	0.96 (0.60)	0.96 (0.58)	0.91 (0.75)	0.761	0.96 (0.49)	0.95 (0.89)	0.961
Cholesterol (mmol/L)	3.50 (1.58)	3.54 (1.63)	2.95 (0.77)	**0.005**	3.56 (1.72)	3.27 (0.91)	0.074
HDL	1.06 (0.45)	1.07 (0.45)	1.00 (0.54)	0.567	1.06 (0.44)	1.07 (0.49)	0.945
LDL	1.96 (1.03)	1.98 (1.05)	1.63 (0.56)	**0.015**	2.00 (1.12)	1.78 (0.55)	**0.035**
HBeAg				0.328			0.886
Negative	246 (86.6%)	226 (85.9%)	20 (95.2%)		194 (87.0%)	52 (85.2%)	
Positive	38 (13.4%)	37 (14.1%)	1 (4.76%)		29 (13.0%)	9 (14.8%)	
Child–Pugh				0.309			0.708
*A*	195 (68.7%)	183 (69.6%)	12 (57.1%)		153 (68.6%)	42 (68.9%)	
*B*	75 (26.4%)	67 (25.5%)	8 (38.1%)		60 (26.9%)	15 (24.6%)	
*C*	14 (4.9%)	13 (4.94%)	1 (4.76%)		10 (4.48%)	4 (6.56%)	
MELD	9.87 (8.59–12.1)	10.6 (2.41)	9.93 (1.86)	0.13	10.6 (2.46)	10.2 (2.03)	0.156

Significance (*P* value) is listed for comparisons between bleeders and nonbleeders at different observation points. BMI: body mass index; baseline LSM: baseline liver stiffness measurements; PVT: portal vein thrombosis; NSBB: nonselective beta-blockers; PT: prothrombin time; TBIL: total bilirubin; ALT: alanine aminotransferase; AST: aspartate aminotransferase; ALP: alkaline phosphatase; GGT: glutamyl transpeptidase; BUN: blood urea nitrogen; HDL: high-density lipoprotein; LDL: low-density lipoprotein; HBeAg: hepatitis B virus *e* antigen; MELD: model for end-stage liver disease.

**Table 2:** Univariate and multivariate logistic regression.

	Rebleeding within 6 weeks				Rebleeding within 1 year			
	Univariate analysis		Multivariate analysis		Univariate analysis		Multivariate analysis	
	OR (95% CI)	*P* value	OR (95% CI)	*P* value	OR (95% CI)	*P* value	OR (95% CI)	*P* value
Age	0.989 (0.956–1.023)	0.541			0.983 (0.962–1.005)	0.129		
Gender	0.666 (0.236–1.879)	0.442			0.869 (0.483–1.663)	0.728		
BMI	1.029 (0.893–1.186)	0.691			1.082 (0.988–1.184)	0.088	1.146 (1.031–1.273)	0.011
Baseline LSM	1.038 (0.998–1.078)	0.060			1.041 (1.014–1.070)	0.003	1.042 (1.011–1.075)	0.008
Portal vein diameter	5.931 (1.397–25.191)	0.016			3.020 (1.120–8.149)	0.029		
PTV	1.203 (0.336–4.314)	0.777			1.553 (0.701–3.440)	0.278		
Using NSBB drugs	0.184 (0.069–0.492)	0.010	0.170 (0.062–0.470)	0.01	0.320 (0.179–0.572)	<0.001	0.274 (0.127–0.482)	<0.001
Hemoglobin	0.979 (0.962–0.997)	0.024	0.979 (0.960–0.998)	0.029	0.980 (0.969–0.991)	0.001	0.976 (0.963–0.998)	<0.001
White blood count	0.856 (0.678–1.080)	0.190			0.780 (0.999–0.913)	0.002		
Platelet count	0.982 (0.968–0.996)	0.010	0.985 (0.971–0.999)	0.032	0.987 (0.981–0.994)	<0.001	0.989 (0.982–0.996)	<0.001
PT	1.029 (0.942–1.124)	0.523			1.020 (0.949–1.096)	0.597		
Albumin	0.998 (0.927–1.074)	0.952			1.005 (0.959–1.053)	0.825		
TBIL	1.001 (0.980–1.023)	0.926			1.000 (0.986–1.014)	0.992		
ALT	1.002 (0.995–1.009)	0.561			0.999 (0.993–1.005)	0.752		
AST	1.002 (0.996–1.007)	0.580			0.999 (0.994–1.004)	0.67		
ALP	0.989 (0.977–1.000)	0.059			0.997 (0.994–1.001)	0.129		
GGT	0.992 (0.982–1.003)	0.157			0.998 (0.995–1.001)	0.109		
BUN	0.980 (0.870–1.105)	0.745			0.989 (0.929–1.054)	0.734		
Creatinine	0.996 (0.978–1.015)	0.698			0.989 (0.975–1.003)	0.137		
Sodium	1.019 (0.950–1.093)	0.604			1.001 (0.981–1.022)	0.898		
Potassium	0.974 (0.844–1.125)	0.724			0.999 (0.981–1.018)	0.915		
Triglycerides	0.848 (0.363–1.985)	0.704			0.984 (0.609–1.590)	0.946		
Cholesterol	0.592 (0.345–0.989)	0.045			0.841 (0.650–1.089)	0.189		
HDL	0.697 (0.249–1.956)	0.493			1.024 (0.574–1.916)	0.941		
LDL	0.546 (0.228–1.107)	0.096			0.754 (0.525–1.092)	0.136		
HBeAg	0.305 (0.400–2.345)	0.254			1.158 (0.516–2.598)	0.722		

All variables were entered in a forward LR elimination procedure with a *P* value to exit set at >0.10. Empty cells refer to the variables excluded from the multivariable-adjusted logistic regression models. BMI: body mass index; baseline LSM: baseline liver stiffness measurements; PVT: portal vein thrombosis; NSBB: nonselective beta-blockers; PT: prothrombin time; TBIL: total bilirubin; ALT: alanine aminotransferase; AST: aspartate aminotransferase; ALP: alkaline phosphatase; GGT: glutamyl transpeptidase; BUN: blood urea nitrogen; HDL: high-density lipoprotein; LDL: low-density lipoprotein; HBeAg: hepatitis B virus e antigen; MELD: model for end-stage liver disease.

**Table 3:** LR and XGBoost models performance parameters.

Models	AUC	Sensitivity	Specificity	Accuracy	Precision	Recall	*F*1
LR (6 W)	0.828 (0.759–0.897)	1.000	0.57	0.602 (0.543–0.660)	0.157	1.000	0.271
LR (1 Y)	0.799 (0.738–0.860)	0.623	0.848	0.799 (0.748–0.844)	0.528	0.623	0.589
XGBoost (6 W)	0.985 (0.907–0.731)	1.000	0.907	0.914 (0.866–0.949)	0.485	1.000	0.653
XGBoost (1 Y)	0.931 (0.806–0.935)	0.957	0.814	0.849 (0.791–0.895)	0.616	0.957	0.750

LR (6 W): logistic regression model predicts rebleeding within 6 weeks; LR (1 Y): logistic regression model predicts rebleeding within 1 year; XGBoost (6 W): XGBoost model predicts rebleeding within 6 weeks; XGBoost (1 Y): XGBoost model predicts rebleeding within 1 year.

## Data Availability

The data used to support the findings of this study are available from the corresponding author upon reasonable request.

## Supplementary Materials

Supplementary Materials Please refer to the supplementary materials for detailed information on secondary prevention measures, liver stiffness measurement, and clinical data collection.
