# Supplementary material for: A Practical Model for Predicting Esophageal Variceal Rebleeding in Patients with Hepatitis B-Associated Cirrhosis
Source: Int J Clin Pract. 2023 Aug 3;2023:9701841. doi: 10.1155/2023/9701841 (PMC10415078; doi:10.1155/2023/9701841)
Supplement: Supplementary Materials — Please refer to the supplementary materials for detailed information on secondary prevention measures, liver stiffness measurement, and clinical data collection. [file 9701841.f1.docx]

**Secondary prevention**

For patients receiving NSBB treatment, either carvedilol or propranolol was used. Carvedilol was started at an initial dose of 6.25 mg once daily and adjusted gradually to the maximum tolerated dose, keeping the heart rate >55 beats per minute and systolic blood pressure >90 mmHg. Propranolol was started at an initial dose of 10 mg three times daily and adjusted gradually to the maximum tolerated dose, keeping the heart rate at>55 beats per minute and the systolic blood pressure >90 mmHg. Patients with first bleeding were treated by EVL in the first endoscopic approach. EVL was performed using commercial multiband devices under sedation with propofol. The varices were ligated from the cardia to the oral side.

**liver stiffness measurement**

The liver stiffness measurements were completed within 1 week after the patient underwent EVL for acute bleeding. For patients with ascites at the time of admission, the LSM was measured after the ascites subsided. Transient elastography was performed with FibroScan (Echosens, Paris, France) using the standard-probe. LSM was considered reliable only if 10 successful acquisitions were obtained and the ratio of the interquartile range over the median (IQR/LSM) was ≤0.3. LSM was expressed in kilopascals. Patients with unreliable LSM results had the examination repeated immediately, and the results were not analyzed if they remained unreliable. The operators were blinded to all clinical data and the diagnoses of the patients.

**clinical data collection**

All patient demographic information, laboratory information, imaging information and or histopathological information were obtained from the electronic medical record of the First Affiliated Hospital of Nanchang University. The main variables collected were name, contact information, gender, age, height, weight, LSM, portal vein diameter, portal vein thrombosis, NSBB drug use, hemoglobin, leukocytes, platelets, prothrombin time, albumin, TBIL, ALT, AST, ALP, GGT, urea nitrogen, blood creatinine, blood sodium, blood potassium, cholesterol, triglycerides, LDL, HDL, and hepatitis B e antigen, endoscopic esophageal varices. In patients with hepatocellular carcinoma, the pathology report was also recorded. Child-Pugh classification and MELD score were calculated from the above indicators. The data were collected independently by two physicians, and checked by a third person.
